# 'Memory and molecular turnover,’ 30 years after inception

**DOI:** 10.1186/1756-8935-7-37

**Published:** 2014-12-09

**Authors:** Richard B Meagher

**Affiliations:** Genetics Department, University of Georgia, Athens, GA 30602 USA

**Keywords:** Methylcytosine, Hydroxymethylcytosine, Post-translational modification, Histones, Acetylation, Nucleosomes

## Abstract

In 1984 Sir Francis Crick hypothesized that memory is recorded in the brain as reversible modifications to DNA and protein, but acknowledged that most biomolecules turn over too rapidly to account for long-term memories. To accommodate this possible paradox he modeled an enzymatic mechanism to maintain modifications on hemi-modified multimeric symmetrical molecules. While studies on the turnover of chromatin modifications that may be involved in memory are in their infancy, an exploration of his model in the light of modern epigenetics produced somewhat surprising results. The molecular turnover rates for two classes of chromatin modifications believed to record and store durable memories were approximated from experiments using diverse approaches and were found to be remarkably short. The half-lives of DNA cytosine methylation and post-translationally modified nucleosomal histones are measured in hours and minutes, respectively, for a subset of sites on chromatin controlling gene expression. It appears likely that the turnover of DNA methylation in the brain and in neurons, in particular, is even more rapid than in other cell types and organs, perhaps accommodating neuronal plasticity, learning, and memory. The machinery responsible for the rapid turnover of DNA methylation and nucleosomal histone modifications is highly complex, partially redundant, and appears to act in a sequence specific manner. Molecular symmetry plays an important part in maintaining site-specific turnover, but its particular role in memory maintenance is unknown. Elucidating Crick’s paradox, the contradiction between rapid molecular turnover of modified biomolecules and long-term memory storage, appears fundamental to understanding cognitive function and neurodegenerative disease.

## Review

### Chromatin modifications record memories and regulate synaptic strength

Thirty years ago, in a letter to *Nature* ('Memory and molecular turnover’), Crick [1] hypothesized that memory is 'stored in the brain’ as reversible modifications to DNA and protein that alter 'synaptic strength’. Only in the last decade has it become clear that changes to the epigenome, modifications to chromatin, such as the 5′ methylation (5Me) of DNA cytosine (C) residues, the post-translational modification (PTM) of nucleosomal histones (for example, acetylation, methylation, phosphorylation), and histone variant specific nucleosome positioning play central roles in memory formation and maintenance and in synapse development [2–5]. Over 100 enzymes and dozens of macromolecular machines have been identified that control the formation and turnover of chromatin modifications and the critical movement of modified nucleosomes within and between gene sequences. To date, only a few of these alterations to chromatin have been definitively linked to memory formation and maintenance or to the development of neural structures. Several diseases leading to cognitive dysfunction have been associated with genetic defects in epigenetic controls [2, 6, 7]. Crick’s early insight, suggesting that secondary modifications to DNA and protein are important to memory formation and storage, has been widely cited.

### The roles of molecular symmetry and turnover rates to memory duration

**Figure 1:**
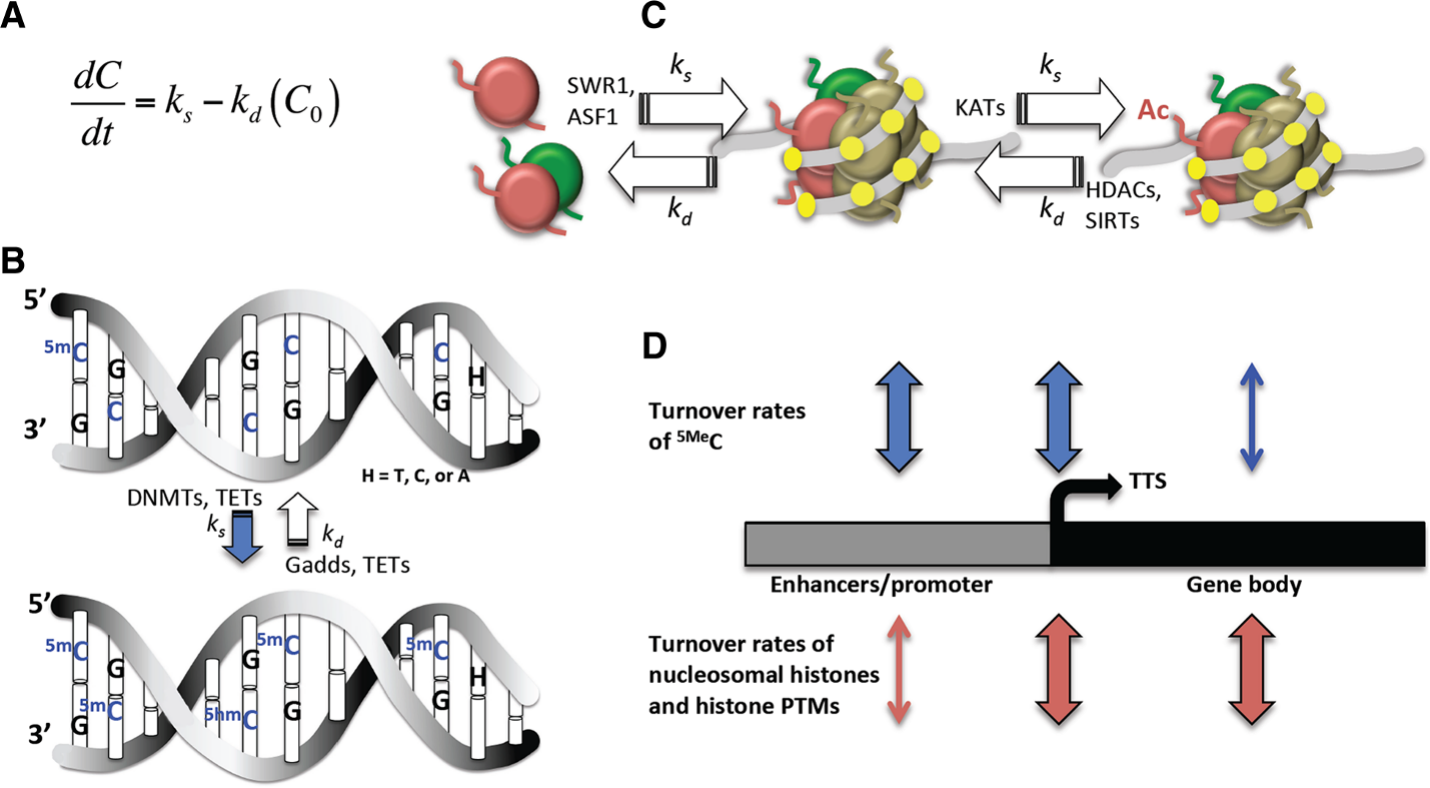
**The rapid turnover of DNA cytosine methylation and nucleosomal histones. (A)** The turnover rate equation. *dC/dt* represents the turnover rate of DNA cytosine methylation or nucleosomal histone PTMs, where C is concentration, t is time, *k*_*s*_ the synthesis rate, *k*_*d*_ the decay rate, and *C*_*o*_ the starting concentration. Approximate turnover rates  (see text) are estimated from the rate of change in modification levels, when neither *k*_*s*_ nor *k*_*d*_ = 0. Half-life (*t*_*1/2*_) is the time it takes for a two-fold change in *C*_*o*_. **(B)** The turnover of DNA 5'-methylcytosine and 5'-hydroxymethylcytosine. The half-life (*t*_*½*_) for ^5Me^C residues is estimated in hours or less at selected sites. DNA methyltransferases (DNMTs) contribute to synthesis of ^5m^C from C, the TETs oxidize ^5Me^C to ^5hm^C and other products discussed in the text, and the Gadds and TETs contribute to the decay of ^5Me^C and ^5hm^C back to C. **(C)** The turnover of nucleosomal histones and histone PTMs. The half-lives for nucleosomal histones and their PTMs are estimated in tens of minutes or less at selected sites. Nucleosomal histone turnover (left) is catalyzed by chromatin remodeling factors such as SWR1 and ASF1. The turnover of acetylated histone side chains (right) is catalyzed by lysine acetyltransferases (KATs) that add acetyl groups and histone deacetylases (for example, HDACs, SIRT1) that remove acetyl groups. The nucleosome is a symmetrical structure composed of four pairs of histones (large spheres) wrapped in approximately 147 bp of DNA (grey line). DNA dinucleotides that may contribute to nucleosomal positioning and CDI are spaced 10 bp apart (yellow dots). **(D)** The turnover of chromatin modifications is site specific with rates varying across gene regions, as indicated by the widths of the arrows.

Crick expressed particular concern that most known biomolecules 'turn over in a matter of days, weeks or at the most a few months’ too rapid to account for long-term memories that might last 'tens of years’. To accommodate this apparent weakness in his theory that remote memory was stored in modified DNA and protein, he modeled an enzymatic mechanism for the long-term maintenance of memory based on the modification of multimeric symmetric molecules, akin to the conservation of symmetrically methylated cytosine DNA residues (^5Me^C) in a CG sequence context (Figure 1). In his example, site-specific DNA methyltransferases (DNMTs) recognize an unmodified cytosine base at a hemi-modified site in the antiparallel complementary strands of DNA and modify it, converting ^5Me^CG/GC to ^5Me^CG/G^5Me^C [8]. Crick’s model highlighting the importance of rapid molecular turnover and molecular symmetry to memory duration has, with few exceptions, been overlooked in the literature [3, 9, 10]. Beyond memory, this model addresses the broader issue of how any cell in any organ maintains its stable identity if its individuality is determined by protein and nucleic acid modifications that turn over rapidly.

### The turnover of DNA cytosine methylation

DNA methylation does indeed contribute to memory formation and maintenance and is required for long-term potentiation of neurons [3, 4, 9, 10]. DNA methylation is considered to be one of the more stable chromatin modifications and one of the few, which once newly established, may even contribute to multi-generationally inherited phenotypes and pathologies [11]. Furthermore, DNA methylation is often phylogenetically conserved among recently duplicated gene sequences. Hence, it seems counterintuitive to consider the rapid turnover of cytosine methylation. Complex machinery is involved in the dynamic maintenance of methylation and has been moderately well characterized, some in relation to learning and memory. For example, the DNA cytosine-5-methyltransferases DNMT1, DNMT3a, and DNMT3b appear to play a complementary and/or overlapping roles in maintenance and in establishing new methylation patterns involved in learning and memory and in storing memories [9, 12, 13]. DNMTs require the antiparallel symmetry of DNA for their activity (Figure 1B). Non-coding RNAs template *de novo* cytosine methylation and assist in recruiting DNMTs and RNA-directed DNA methylation machinery (for example, DRM1, DRM2, CMT3) to methylation sites [14–16].

The chemical turnover rates for DNA ^5Me^C may be approximated from pioneering studies that assayed dynamic changes in steady state ^5Me^C levels over relatively short time periods. It is useful to recall that the turnover rate of a molecule  is equal to the synthesis rate (*k*_*s*_) minus the decay rate (*k*_*d*_) multiplied by the starting concentration (*C*_*o*_) or  (Figure 1A) [17]. The half-life (*t*_*1/2*_) of a molecule is the time it takes for *C*_*o*_ to increase or decrease two-fold, when the change in concentration is dependent only upon synthesis or decay. Most reports of dynamic changes to ^5me^C levels set neither the synthesis *k*_*s*_ nor decay *k*_*d*_ rates to zero, and hence, only approximate the rate of turnover  or half-life (*t*_*1/2*_~). An early indication that DNA methylation regulating relevant gene expression might turn over rapidly comes from a study of changes in the rat hippocampal CA1 region 1 h following context dependent fear conditioning [13]. *DNMT3a* and *DNMT3b* transcripts increase significantly. Methylation of a CG island (CGI) in the *PP1C* promoter increases 45-fold, while methylation of a CGI in the *RELN* promoter decreases two-fold within this hour. There is an accompanying reduction in *PP1C* and increase in *RELN* transcript levels. Hence, the approximate turnover rate for ^5Me^C in these two GCIs is 1 h or less. The levels of methylation of both promoters returned to base line 24 h after training. The chemical inhibitor AzaC prevents remethylation by being incorporated into DNA and inhibiting the activity of DNA methyltransferases. Hence, AzaC treatments set the synthesis rate at incorporated sites near to zero so that turnover rates may be more accurately measured . Hippocampal infusion with limited amounts of AzaC immediately after training completely blocks contextual fear conditioning, greatly reduces the methylation of these two promoter CGIs, and reduces *Reelin* promoter methylation even lower than with fear conditioning alone. AzaC infusion also reverses changes in *PP1* and *Reelin* transcript levels. This experiment confirms that methylation levels in these CGIs are highly dynamic and suggests turnover rates of less than 1 h. Subsequent studies have further supported the rapid turnover of DNA methylation for a subset of C residues. When AzaC is added to non-dividing cultured cells, 10% of total ^5Me^C in the genome is lost after 2 h, but the level of loss increases to only 13% after 48 h of treatment, suggesting that only a fraction of total ^5Me^C sites are turning over rapidly [18].

AzaC inhibitor studies may be criticized as increasing rates of DNA repair and perhaps the loss of ^5Me^C by mechanisms not necessarily relevant to normal turnover [19]. Alternative estimates of ^5Me^C minimal turnover rates may be made, independent of the use of DNA methylation inhibitors, based on the approximately hour-long cycles of *pS2/TFF1* transcript expression observed when this gene is induced in multiple cell types [20]. Hour-long cycles of increased and decreased DNMT3a and 3b expression are in phase with decreases and increases in *pS2* transcript levels. There are corresponding hour-long cycles of methylation and demethylation at eight out of 19 specific CG sites assayed in the *pS2* promoter, which correlate with loss and gain of *pS2* transcripts, respectively. Methylation levels change several-fold during each hourly cycle. As methylation levels increase dramatically for 15 to 20 min of each cycle one may presume *k*_*s*_ is at a maximum and *k*_*d*_ at a minimum during this period. Hence, the approximate turnover rate  at eight particular ^5Me^C residues in the *pS2* promoter region may be estimated to be significantly less than 10 min. In summary, the turnover of a subset of ^5Me^C residues appears to be extremely rapid, particularly in some promoter regions (Figure 1D), validating Crick’s concerns for one class of molecules that are involved in maintaining durable memories. The mechanisms that identify the DNA sequence context of the ^5Me^C sites that turn over most rapidly remains an ongoing area of research.

The machinery responsible for the predominant DNA ^5Me^C de-methylation activities contributing to turnover has been difficult to pin down in any tissue, let alone de-methylation in the brain, which might impact memory duration [21]. There are three phylogenetically-related members in a family of non-enzymatic growth suppressors, the growth arrest and DNA-damage-inducible Gadd proteins (Gadd45a, -45b and -45 g). All three are associated with double stranded DNA repair machinery and are expressed in the brain [22]. Gadd45a was the first member to be linked to DNA demethylation, when it was isolated during a screen for embryonic cDNAs capable of activating co-transfected methylation-silenced gene encoding a SV40 early promoter-luciferase reporter [23]. Subsequent assays show that the transient overexpression of Gadd45a activity in HEK293T cells along with methylation-sensitive *SV40*, *Xbra*, *BRE*, or *TOP* promoter driven reporters result in the ^5Me^CG demethylation of the promoters and increased expression of all four reporters within the 24 h assay [23]. The results on reporter de-methylation parallel those using co-treatment with AzaC or expression of the same reporters in *DNMT1-/-* cells, except that Gadd45a expression further activates unmethylated as well as methylated reporters, while AzaC only activates methylated reporters. This suggests that Gadd45a activity not only contributes to the de-methylation of fully symmetrically methylated DNA (Figure 1C), but may also overcome rapid endogenous re-methylation of C to ^5Me^C in a fully unmethylated context that affects the dynamics of promoter activation and re-silencing.

The relative importance of the Gadds to the decay of DNA methylation was challenged by two studies, in particular. First, the transient expression of Gadd45a in cultured cells did not de-methylate or activate a methylated version of a methylation-sensitive *Oct4/Pou5f1* promoter driven reporter transgene in HEK293T cells [21, 24]. Second, mice lacking the Gadd45a variant show relatively normal development and normal methylation levels of the endogenous *Oct4* promoter DNA and of global DNA [25]. However, a more recent study in embryonic stem (ES) cells suggests that Gadd45a acts in conjunction with other machinery to achieve demethylation on a subset of sequences, such as those in the *Oct4* promoter. The demethylation and activation of methylation silenced *TK* and *Aprt* promoter-reporter constructs and endogenous pluripotency genes (Oct4/*Pou5f1, Nanog*) require the co-expression of four genes involved in demethylation, *Gadd45a*, *Tet1*, *Aid*, and *Mbd4* [26]. Silencing any one of these four genes abolished 50% to 99% of the demethylating activity necessary to activate each of the four promoters. These data support the view that the Gadds are an essential part of complex multifaceted machinery involved in the sequence-specific control of DNA cytosine de-methylation and turnover. Yet, there are many unanswered questions and problems to be addressed concerning the role of Gadds in ^5Me^C turnover. The three Gadd family variants are likely partially redundant or overlapping in function, but the phenotypes of double and triple Gadd mutants have not been reported. Turnover appears to be rapid, yet none of these studies examined Gadd45a expression constructs co-transfected with hemi-methylated reporters, in order to assay the contribution of endogenous remethylation and silencing to turnover rates after methylcytosine is removed from one strand of a ^5Me^CG/G^5Me^C sequence. Nor are their reports of the role of Gadds in the turnover of ^5Me^C in the asymmetric CH dinucleotide context, where H is T, C, or A (Figure 1B). Gene-region-specific ^5Me^CH dinucleotide levels appear to be particularly well correlated with neuron-specific gene expression and the development of synaptic density in the human and mouse brain cortex [27].

There is initial evidence that three Gadds impact neural activity in the brain. Electroconvulsive treatment (ECT) induces proliferation of neural progenitors and dendritic development. ECT also induces the demethylation of specific ^5Me^CG dinucleotides in the *BDNF* and *FGF-B1* promoters in neural cells of the dentate gyrus of the hippocampus, but not measurable global DNA demethyation [28]. The approximate two-fold decrease in methylation of several CG dinucleotides in the *BDNF* and *FGF-B1* promoters, which follows 4 h after ECT, was abolished in *Gadd45b-/-* mice. Transient 25- and eight-fold increases in expression of *Gadd45b* and -*45 g* transcripts*,* respectively, peaked 1 h after ECT, but returned to base line levels 4 h post ECT. This suggests that Gadd45b and -45 g may contribute to a dynamic demethylation response in the brain with the half-lives of some of the targeted ^5Me^C residues measured in hours. The levels of *Gadd45a* transcripts did not change with ECT. In addition, further evidence for the dynamic role of Gadds in methylation turnover in neural cells comes from their co-expression with DNMTs. When *Danio rerio* retinal Müller glia transition from a quiescent supportive cells to multipotent neural progenitor cells (NPCs) following brain injury, homologs of *Gadd45a* and *Gadd45g* and four *DNMTs* (*DNMT1*, *4*, *5*, *7*) are concomitantly induced [29]. Similarly, mouse brain NeuN-High neuronal cell nuclei with decondensed chromatin not only express elevated levels of transcripts involved in learning and memory and multipotency, but they also more highly express transcripts for *Gadd45a* and *-45b* and *DNMT1* and *3A*, relative to less epigenetically active normal-sized NeuN-Low neuronal cell nuclei [30]. In summary, the role of the Gadd family of repair proteins in the turnover of DNA methylation and memory duration remains an important problem worthy of further study, in spite of the apparent complexity of various published reports.

Recently the *ten-eleven-translocation (TET)* gene family (*TET1*, *TET2*, *TET3*) has emerged as central to removing ^5Me^C residues [21]. TETs are DNA dioxygenases that catalyze the conversion of ^5Me^C to another biologically active form 5′-hydroxymethylcytosine (^5hm^C) and require a double stranded β-helix for their activity. TETs have the potential go on to oxidize ^5hm^C first to 5′-formylcytosine (^5f^C) and then to 5′-carboxylcytosine (^5ca^C). They perform all three reactions efficiently on ^5Me^C modified DNA templates *in vitro*, but *in vivo*^5hm^C accumulates to much higher levels than the latter two products. DNA repair enzymes convert ^5ca^C to C completing the turnover of ^5Me^C. TET generated ^5hm^Cs in human ES cells and the mouse fetal and adult brain cortex are found almost exclusively in a CG context [27, 31]. Thus, TETs have the potential to impact the turnover of a large subset of ^5Me^C residues, but are unlikely to impact the large fraction of ^5Me^C residues in the CH dinucleotide context. As an example of the potential of TETs to impact general methylation, cultured primary cells from benign smooth muscle leiomyoma tumors overexpress Tet1 and Tet3. They show increased global DNA ^5hm^C relative to cultured primary myometrial cell controls. Partial RNAi silencing of *Tet1* or *Tet3* expression for 24 h reduces global DNA ^5hm^C by only 25%, but greatly reduced the growth rate of leiomyoma cells [32]. As to the sequence specificity of TET activity, Tet1 binds in or proximal to the transcriptional start sites of 6,573 promoter sequences in ES cells, more than 85% of which were rich in CG dinucleotides and most of these were also enriched for ^5Me^C and ^5hm^C [33]. A more recent report shows that Tet1 activity results in the accumulation of ^5hm^C at the edges of methylation rich CGIs, preventing the spread of methylation into normally unmethylated CG rich regions [34]. Taken together current evidence suggests that TETs act preferentially on a subset of ^5Me^C residues concentrated in promoter regions. Tet1’s demethylating activity appears to prevent aberrant methylation and silencing of many CG rich promoters to maintain appropriate levels of transcription.

All three TETs are well expressed in the brain and the importance of their synthesizing ^5hm^C is only recently being understood. ^5hm^C levels are three-fold to 50-fold higher in some parts of the brain such as the cerebral cortex, than in most other organs [21, 35]. The levels of ^5hm^C are estimated at 40% of the ^5Me^C levels in this region of the brain. In Purkinje and granule cell neurons in the brain ^5hm^C is estimated to be 0.6% and 0.2% of total nucleotides, respectively [36]. Tet1 deficiency is associated with defects in neural development, altered expression of neuronal transcripts, and deficiencies in spatial learning and memory. For example, Tet1 knockout mice show a loss of memory extinction and reduced expression of critical neuronal regulated transcripts *Arc*, *Fos*, and *Npas4* in the cortex and hippocampus [6]. CG dinucleotides in the *Npas4* promoter, suspected to regulate its transcriptional activity, are hyper-methylated in Tet1 knockout mice. While ^5hm^C levels are low in the cerebellum and hippocampus of 7-day-old mice, the levels of ^5hm^C increase two- to five-fold, respectively, in the 6-week-old and adult mice [37]. In these later stages almost half of modified cytosines in CG context in these regions of brain are ^5hm^C. Other studies found that the levels of ^5hm^C are low in the mouse brain cortex at conception, but increase rapidly to near adult levels 6 weeks later, increasing concordantly with increases in synaptogenesis [27, 37]. Finally, in the fetal mouse brain Tet2 activity appears necessary to hydroxymethylate the few percent of ^5Me^C residues destined to be demethylated at later states in development [27]. The tantalizing implication from these studies is that the TET-enzyme activity and ^5hm^C-dependent turnover rate of ^5Me^C may be higher in the brain to assist with more rapid responses of the neuronal methylome to learning and memory. But these results only increase the concern for Crick’s paradox, because more rapid TET-dependent turnover works against ^5Me^C-dependent memory duration.

### The turnover of nucleosomal histone side chain modifications

Gene-specific nucleosomal histone PTMs are involved in memory formation, sometimes changing in a matter of minutes after a stimulus, and also contributing to durable memory formation [3, 4]. There is only limited evidence that gene-specific histone PTMs are multi-generationally inherited and PTMs are rarely phylogenetically inherited among duplicated regions of DNA [11]. This suggests PTMs are more transient than DNA methylation and might turnover more rapidly. Turnover rates for nucleosomal histone variants and by default, histone PTMs, were approximated from experiments that examined changes in their levels over short time periods. A pioneering report in 1990 examined the incorporation of tritiated acetate into the major histone variants (H2A, H2B, H3, H4) in dividing cells without and with the addition of the HDAC inhibitor trichostatin A (TSA) [38]. This and subsequent related pulse-chase labeling experiments using HDAC inhibitors estimated the half-lives (*t½*) of acetylated histone variants are in the range of 2 to 40 min  [39]. Another set of turnover rate measurements were made independent of the use of inhibitors or isotope incorporation in yeast with cells arrested in the G1 phase of growth. Turnover rate estimates may be made based on the approach-to-steady-state increases of histone incorporation into nucleosomes, after induction of transgenes expressing epitope tagged histone variants . These studies are limited by the rate at which tagged variants are newly synthesized and reach a high enough steady state levels to compete maximally with endogenous histone variants. The rate of tagged histone H2b incorporation into nucleosomes suggests a turnover rate of less than 30 min for nucleosomes associated with both transcriptionally active and inactive genes [40]. Similarly, rate of incorporation of tagged histone H3 into nucleosomes, suggests half-life of the H3 variant may be less than 10 min for the 'hottest’ most actively replaced nucleosomes relative to H3 incorporation into other nucleosomes, which turn over with half-lives estimated in hours or longer [41]. These studies also suggest that in yeast nucleosomal histone turnover rates were relatively low in actively transcribed gene regions, but high in the proximal regions of promoters.

A genome wide steady state measurement of histone variant turnover rates  was made in cultured insect cells independent of transgene induction lag times using the methionine analog azidohomoalanine (Aha). Aha is rapidly incorporated into protein after its addition to cell culture media [42]. After non-dividing late log phase cells are given short pulses of Aha, nucleosomes are purified, and nucleosomal Aha is specifically chemically coupled to biotin. Newly made biotin-tagged nucleosomes are affinity purified and associated DNA quantified across the genome. Because the levels of nucleosomal Aha labeling of gene-region specific DNA sequences in active genes appears nearly complete after a 20 min pulse of Aha, the half-life (t_½_) for histone proteins within nucleosomes in these gene regions may estimated to be less than 10 to 20 min, but may occur more slowly in flanking promoter/enhancer regions (Figure 1D). In summary, the discordance between the short half-lives of this major class of modified biomolecules implicated in memory formation and memory duration is extreme.

Following the symmetry argument in Crick’s model for maintaining modifications, the symmetrical pairing of the four core histones (for example, two histone H4 subunits, two H2b subunits, and so on) in a nucleosome might direct the re-modification of hemi-modified histone pairs in nucleosomes. A model utilizing nucleosomal symmetry analogous to that for the activity of DNMT1 on hemimethylated DNA is imagined. However, one such study shows that the symmetrical pairing of differentially modified H4 subunits accounts for maintaining only a small fraction of the two histone H4 PTMs assayed, H4K20me2 and H4K20me3 [43]. Hence, nucleosomal symmetry may represent only a partial solution to the problem of histone PTM maintenance. Moreover, no such measurements taking into account the symmetry of PTMs have been reported for the brain.

Complex models for the deposition and turnover of nucleosomes with particular modified histone compositions have been proposed that preserve their position in chromatin, a process known as chromatin domain inheritance (CDI). CDI is of necessity linked to DNA replication, DNA repair, and transcription, but must be considered in any nucleosomal histone turnover model. Because it is hard to imagine a complete physical mechanism for the CDI of properly modified nucleosomes following replication or transcription, it is reassuring that numerous proteins and multiprotein complexes contributing to replicative CDI have been identified, including proliferating cell nuclear antigen, mini-chromosome maintenance complex, the histone chaperone anti-silencing factor 1 (ASF1), chromatin assembly factor 1, and histone variant exchange complexes (for example, SWR1) [44–47]. As an example activity, histones H3 and H4 bound to ASF1 are deposited on both strands of the newly replicated DNA followed by H2A and H2B. Yet, in spite of progress understanding some of the mechanics of histone and nucleosome deposition and conservation beyond a DNA replication fork, the precise factors that determine CDI and how this entire process impacts durable memory-related gene expression remain illusive.

One major problem for understanding CDI has been that chromatin domains for nucleosomes were not previously thought of as DNA-sequence specific, and hence, the antiparallel symmetry of DNA did not seem relevant. However, a consensus rotational palindromic repeat of 10.5 bp (R-YYYYYRRRRR-Y, R = purine, Y = pyrimidine) that bends correctly around and binds the eight core nucleosomal histones was revealed using advanced computational methods to examine tens of thousands of nucleosome-delimited 147 base pair (bp) DNA sequences from yeast, plants, and humans [48]. Typically on the order of several to 10 dinucleotides from among the 14 repeats of the DNA double helix have the correct sequence and orientation to bind and position the nucleosome (yellow dots, Figure 1C) [49]. Because DNA residues are contacting particular histone subunit amino acid residues, the exact histone variant composition and perhaps histone PTMs in the nucleosome appear to determine the sequence-specificity for nucleosome binding and positioning. For example, nucleosomes containing the histone variant H2AZ that are enriched arround transcriptional start sites and nucleosomes containing the centromeric histone 3 (CENH3) that are highly enriched within centromeres each contact different dinucleotide consensus sequences [11, 50, 51]. In a recent study, Zovkic *et al.* [5] show that there is a rapid exchange of the two H2AZ variant nucleosomes immediately flanking the transcription start site of memory-related genes in the CA1 region of the hippocampus following fear conditioning (that is, remote memory and memory consolidation) [5]. In short, one key to the CDI of nucleosome specific histone PTMs associated with genes involved in memory maintenance may lie in the symmetrical double-strand DNA sequence-specific code for nucleosome positioning.

### Current limitations and future studies on memory and molecular turnover

It is worth noting that most of the literature on the turnover of chromatin structures does not come from the CNS. With few exceptions, the examples measuring molecular turnover associated with direct measurements of memory acquisition, consolidation, remote (long-term) memory, and extinction come from the best-studied model system for learning-related plasticity, the hippocampus. Yet, the latter of these memory processes involve a dialog with neurons in other regions of the brain such as the neocortex [52, 53]. Linked cortical neurons are undoubtedly being reprogrammed through dynamic changes in epitype that are not being measured. Perhaps the extension of new technologies, such as the fluorescence activated nuclear sorting or isolation of nuclei tagged in specific cell types (INTACT) in the brain will enable the analysis of turnover in different classes of neurons from multiple regions of the brain [27, 30, 54].

Also beyond the scope of current research is the dissecting possibility that the thousands of synaptic connections formed by one neuron are recorded or programmed as combinatorial genome-wide changes to numerous different chromatin structures that must be maintained or strengthened for remote memories [55]. A cubic millimeter of the mammalian neocortex contains nearly a hundred thousand neurons with as many as an hundred million synapses [56], whose synaptic strength must be rapidly regulated. It is hard to conceive that changes in epitype in the nucleus of each neuron direct such complexity, even if the combinatorial capacity exists in the epigenome. Non-chromatin associated decentralized epigenetic phenomena such as the localization of mRNAs (for example, MKK7, PKMζ) and non-coding RNAs (for example, miR-124) contribute to neurite- and synapse-specific gene expression [57–59]. Further, memory-related transcriptional regulatory proteins (for example, CRTC1) may be localized near synapses and subsequently transported to the nucleus to couple synaptic transmission with transcription [60, 61]. Yet, RNA and protein transport and their cytoplasmic positioning for remote memory may be programmed by chromatin-modifications, modifications that must be maintained for some period and are subject to molecular turnover. Such unresolved issues highlight the early pioneering stage of research on the epigenetics of long-term memory.

## Conclusions

Crick’s model highlighting the importance of molecular turnover and the role of molecular symmetry to memory duration appears to have been way ahead of its time and is worthy of serious consideration as we explore the molecular bases of memory. Loss of normal epigenetic control and defects in remote memory are part of most cognitive disorders, including Alzheimer’s disease, Rett syndrome, Rubinstein-Taybi syndrome, Prader-Willi syndrome, Schizophrenia, Fragile X mental retardation, and major depression [6, 7, 62]. Defects in histone PTMs and DNA methylation have been strongly implicated in the loss of cognitive function and memory and most of these disorders. Beyond the role of symmetry in CG methylation, little is known about the contribution of molecular symmetry to maintaining modifications in relevant protein complexes such as the nucleosome. Closing the gap between understanding memory duration and the maintenance of chromatin modifications in the face of rapid molecular turnover appears paramount to the study of neurobiology and neurodegenerative disease.

### General abbreviations

AzaC (5-aza-2′-deoxycytidine) Aha (azidohomoalanine), bp (base pair), BER (base excision repair), CDI (chromatin domain inheritance), CGI (CG rich Islands), CNS (central nervous system), ECT (Electroconvulsive treatment), ES cells (embryonic stem cells), HEK293T cells (human embryonic kidney 293 cells), histone PTM (histone post-translational modification), INTACT (isolation of nuclei tagged in specific cell types) [63, 64], NPC (neural progenitor cell), ^5Me^C (5′-methylcytosine), ^5hm^C (5′-hydroxymethylcytosine), ^5f^C (5′-formylcytosine), ^5ca^C (5′-carboxylcytosine), RNAi (RNA interference), TDG (thymine-DNA glycosylase).

### Protein/gene abbreviations

Aid/AICDA (Activation-Induced Cytidine Deaminase) Aprt (adenine phosphoribosyltransferase), APC/PPP1R46 (Adenomatous Polyposis Coli), ARC (Activity-regulated cytoskeleton-associated protein), ASF1 (histone chaperone anti-silencing factor 1), BDNF (Brain-derived neurotrophic factor), BRE/BRACC45 (Brain And Reproductive Organ-Expressed), BRN2/ POU3F2 (Brain-specific 2/N gene, POU domain class 3 homeobox 2), CENH3 (centromeric histone 3), CRTC1 (CREB-regulated transcriptional coactivator), CRYAA/CRYA1 (Crystallin, Alpha A), DAZ1/SPGY (Deleted In Azoospermia 1), DNMTs (DNA methyltransferases 1, 3A, 3B from mammals and 1 4, 5, 7 from fish), DRM1, DRM2, CMT3 (RNA directed de novo DNA methyltransferases), FGF-B1/FGF2 (Fibroblast growth factor 1 basic), Fos (FBJ Murine Osteosarcoma Viral Oncogene Homolog), GADD45a, GADD45β and GADD45g (DNA cytosine demethylases, Growth arrest DNA damage inducible protein isoforms), HDAC (histone deacetylase), H2AZ (histone 2a, isoform Z), Mbd4/MED1 (Methyl-CpG Binding Domain Protein 4), MeCP2 (methyl-CpG binding protein 2), MKK7 (mitogen-activated protein kinase kinase 7), MYD118 (myeloid differentiation primary response factor), microRNA 124 (miR-124), Npas4 (Neuronal PAS Domain Protein 4), NeuN/RBFOX3 (Neuronal nuclei, Hexaribonucleotide Binding Protein 3), OCT4/POU5F1 (Octamer-binding transcription factor 4, PKMζ (protein kinase M zeta), POU domain, class 5, transcription factor 1), PP1C/PP1Cγ/PPP1G (serine threonine protein phosphatase 1, gamma subunit), pS2/BEC1 (Breast Cancer Estrogen-Inducible Protein, Trefoil Factor 1), RELN (Reelin), SV40 (simian vacuolating virus 40), TET1, 2, 3 (ten-eleven-translocation DNA dioxygenases), TK1 (Thymidine kinase 1), TOP1 (Topoisomerase DNA 1), Xbra (Xenopus brachyury).
